# GSA and BIGD: Filling the Gap of Bioinformatics Resource and Service in China^*^

**DOI:** 10.1016/j.gpb.2017.02.001

**Published:** 2017-02-22

**Authors:** Jingchu Luo

**Affiliations:** College of Life Sciences, State Key Laboratory of Protein and Plant Gene Research and Center for Bioinformatics, Peking University, Beijing 100871, China

In the 2017 first issue of this Journal – *Genomes, Proteomes and Bioinformatics* – a special database article entitled “GSA: Genome Sequence Archive” [1] is published. This article provides a brief introduction to the platform developed by the authors from the BIG Data Center (BIGD) of Beijing Institute of Genomics (BIG), Chinese Academy of Sciences (CAS). The aim of the GSA project is to collect, integrate, and archive raw sequence data submitted by domestic and international users. It is one of the major activities being carried on by a team of around 50 young bioinformaticians at BIGD. In addition to the GSA system, they are also working on several bioinformatics service-orientated projects as described in one of their recent publications [2].

The past half century has witnessed great advances in molecular biology. The deciphering of the genetic code and the establishment of the central dogma following the discovery of the DNA double helix formed a solid theoretical basis for the field of life sciences. On the other hand, the influential works by Frederick Sanger and others to determine the peptide, tRNA, and DNA sequences, as well as the fundamental endeavor by John Kendrew and Max Perutz to solve the three-dimensional structure of proteins, marked the beginning of the accumulation of molecular biological data.

## Protein sequence databases

The first efforts to collect protein sequence data were made by Margaret Dayhoff, a bioinformatics pioneer at the US National Biomedical Research Foundation (NBRF). In 1965, she published a book “Atlas of protein sequence and structure” with 65 sequences she can find then, and updated it in several new editions in the following years. This is the prototype of the first protein sequence database Protein Information Resource (PIR). Based on the sequences of several protein families, she constructed the amino acid substitution scoring matrix PAM, which is still widely used in sequence alignment and database similarity search. PIR went online in 1984 and can be searched through telephone line (https://en.wikipedia.org/wiki/Margaret_Oakley_Dayhoff). Two years later, Amos Bairoch, a graduate student at the University of Geneva, Switzerland started to annotate protein sequences by manually adding functional and other information into each entry, and created another protein sequence database Swiss-Prot (https://en.wikipedia.org/wiki/Amos_Bairoch).

## Protein structure databases

The first protein structure database Protein Data Bank (PDB) was established in 1971. Different from the protein sequence databases, in terms of the organization history, it was a joint effort by partners from the Europe and the US. As stated in its first announcement as Crystallography News on Nature New Biology [3], the Cambridge Crystallographic Data Center in the UK and the Brookhaven National Laboratory in the US made an agreement to maintain the same copy of the data files, and distribute them freely to the community.

## Nucleotide sequence databases

By the end of the 1970s, Sanger sequencing became a routine work and DNA sequences started to accumulate. It was foresighted by prominent scientists that it was time to set up a nucleotide sequence database since large-scale DNA sequencing was on its way. In 1979, a group of computational biologists headed by Walter Goad at the Los Alamos National Laboratory in the US began to collect nucleotide sequences and developed algorithms to analyze DNA and protein sequences. The popular Smith-Waterman algorithm for local sequence alignment was just one of these tools. The nucleotide sequence database GenBank started to operate in 1982 sponsored by the National Institutes of Health (NIH), together with several other US funding agencies including the National Science Foundation (NSF), the Department of Energy (DOE), and the Department of Defense (DOD). In the same year, the European Molecular Biology Laboratory (EMBL) located in Heidelberg, Germany announced the first release of the European Nucleotide Data Library under the same name of the organization, *i.e.*, EMBL-Bank, or sometimes simply refer to EMBL.

## National Center for Biotechnology Information

By the end of 1980s, the sequence databases of nucleic acids and proteins, as well as the protein structure databases, already contained considerable number of entries, while software tools for analyzing the sequence and structure data became available on mini- and micro-computers. At the same time, the NSFnet, a pilot project of research and education network supported by the NSF started to serve several computing centers in the US. Proposed by the late senator Claude Pepper, the National Center for Biotechnology Information (NCBI) located at the north capital was set up in November, 1988, as a division of the National Library of Medicine. As a biomedical information resource center, NCBI has been serving not only NIH partners, but also the worldwide community of molecular biologists. Under the leadership of David Lipman, the director of the center, NCBI became the largest bioinformatics center in the world. Hundreds of databases and software tools, such as the biomedical abstract database PubMed, the reference sequence database RefSeq, and the database similarity search tool BLAST, are available freely for everyone. In 1989, NCBI started to maintain and distribute GenBank as one of the core data resources.

## European Bioinformatics Institute

The European Bioinformatics Institute (EBI) was created in 1994. It is located in the Wellcome Trust genome campus in south Cambridge, UK. As an outstation of the EMBL, EBI is funded mainly by the European Community and most of the EBI staff are from the European member states. It has been playing a significant role in bioinformatics services for more than 20 years not only for Europe but also for the rest of the world, as the second largest resource center next to NCBI. In addition to the nucleotide database EMBL-Bank, EBI also hosts various databases including the genome repository ENSEMBL, the database of protein families and domains InterPro, and the ontology of gene functions and processes Gene Ontology.

## The international database collaboration

The creation of the NCBI, a US government-supported center, and the EBI sponsored by the European Community secures the long-term sustainability of bioinformatics services at national and international levels. In the meantime, the formation of the international collaboration of three core databases of molecular biology, the nucleotide sequence, the protein sequence, and the protein structure, came into age. In 2003, three partners responsible for the maintenance and distribution of macromolecular structures, PDBe at EBI, PDBj from Japan, and the Research Collaboratory for Structural Bioinformatics (RCSB) PDB in the US, signed an agreement to form a world-wide consortium (http://www.wwpdb.org/). Soon after, the International Nucleotide Sequence Database Collaboration (INSDC, http://www.insdc.org/), another triple alliance originating from GenBank of NCBI, EMBL-Bank of EBI, and the Japanese newcomer DDBJ, was launched in 2005. In the same year, the international universal protein sequence database UniProt was founded (http://www.uniprot.org/). UniProt consists of three components, Swiss-Prot from the Switzerland, PIR from the US, and TrEMBL, a database of automatically translated amino acid sequences from the coding regions of EMBL nucleotide sequences.

## The birth of the Internet and the big data age

The birth of the Internet at the beginning of the 1990s opens a new era, the era of information age. Proposed by Nobel Prize laureate Walter Gilbert in his prospective article published on Nature in January 1991 [4], “*we must hook our individual computers into the worldwide network that gives us access to daily changes in the database and also makes immediate our communications with each other*”. He concluded that biology research had a paradigm shift. A decade later, the release of the two draft human genome sequences generated by two different armies, the government-supported Human Genome Project (HGP) [5] and a private enterprise Celera Genomics [6], marked the new stage of the genomic research. Benefiting from rapid development of the high-throughput and low-cost next-generation sequencing technology, various genomes and metagenomes have been sequenced. According to the latest (by 30 January 2017) Genomes OnLine Database (GOLD, https://gold.jgi.doe.gov/), a total of 265,734 organisms have already been, or are being sequenced [7]. There is no doubt that the big data revolution will influence the molecular biology research greatly in the coming years, and data release and distribution is essential for all research activities [8].

## GSA and BIGD

To meet the need of big data demands, it is of essence to build a national infrastructure for bioinformatics resources and services in China. Unfortunately, it remains as a dream for bio-scientists despite endless advocacy and efforts from two generations of prominent scientists of the field in the last three decades [9]. As we can learn from the above historical overview that China is far behind the developed countries in terms of providing bioinformatics resources and service, and has not been involved in any of the international collaboration until now.

Fortunately, the GSA project is the first step toward this direction. Sequence data generated by around 200 projects from 39 institutions have already been submitted to this system since it went online in December 2015, and it is recognized internationally as indicated by the publication of these projects on several international journals. Moreover, the GSA project is just one of the main activities of the BIGD [2]. Several other projects have been initiated in the past year and are being actively carried on, including several databases from genome to transcriptome, to methylome, and to the genome variation map with a collection of featured animal and plant species. And an international workshop was organized two months ago, speakers from NCBI, the European Molecular Biology Network (EMBNet, http://www.embnet.org/), and other international bioinformatics centers came to exchange experiences on bioinformatics research and services. Most recently, the first meeting of the international scientific advisory board of BIGD was held. The efforts were well appreciated by the field’s experts, such as board members from NCBI, EBI, DDBJ, and other international bioinformatics institutions.

Although the BIGD is still in its infancy, I hope that, it can grow up quickly and healthily, with steady support from governmental funding agencies and from the community, and ultimately become a national bioinformatics center. Finally, I would also like to say a few words borrowed from the UK bioinformatician Alan Bleasby, to the members of the BIGD, “*I don’t think we can get a Nobel prize by what we are doing so, but the Nobel prize winners know what we are doing for*”.

## Competing interests

The author declares no competing interests.
